# Relationship between infarct size and serum uric acid levels during the acute phase of stroke

**DOI:** 10.1371/journal.pone.0219402

**Published:** 2019-07-11

**Authors:** Rodrigo Fernández-Gajardo, José Manuel Matamala, Rodrigo Gutiérrez, Prudencio Lozano, Ignacio A. Cortés-Fuentes, Camilo G. Sotomayor, Gonzalo Bustamante, Juan A. Pasten, Gabriel Vargas, Rodrigo Guerrero, Pablo Reyes, Gabriel Cavada, Walter Feuerhake, Ramón Rodrigo

**Affiliations:** 1 Department of Neurological Sciences, Faculty of Medicine, University of Chile, Santiago, Chile; 2 Biomedical Neuroscience Institute (BNI), Faculty of Medicine, University of Chile, Santiago, Chile; 3 Molecular and Clinical Pharmacology Program, Institute of Biomedical Sciences, Faculty of Medicine, University of Chile, Santiago, Chile; 4 Department of Neuroscience, Faculty of Medicine, University of Chile, Santiago, Chile; 5 Department of Neurology, Clínica Santa María, Santiago, Chile; 6 School of Public Health, University of Chile, Santiago, Chile; Albany Medical College, UNITED STATES

## Abstract

**Introduction:**

Uric acid has gained considerable attention as a potential neuroprotective agent in stroke during the last decades, however, its role in the pathophysiology of ischemic stroke remains poorly understood. A serial evaluation of uric acid levels during the acute phase of stroke and its association with infarct size on magnetic resonance imaging is lacking.

**Methods:**

We present a cohort study of 31 patients with ischemic stroke who were not candidates for thrombolysis according to current criteria at the time. We performed daily measurements of serum uric acid and total antioxidant capacity of plasma during the first week after symptoms onset and 30 days after. Infarct size was determined in the acute phase by a DWI sequence and the final infarct size with a control MRI (FLAIR) at day 30.

**Results:**

Uric acid significantly decreases between days 2 to 6 compared to day 1, after adjustment by sex, age and DWI at diagnosis, with a nadir value at 72h. A mixed model analysis showed a negative association between DWI at diagnosis and uric acid evolution during the first week after stroke. Moreover, multivariable linear regression of uric acid values during follow up on DWI volumes demonstrated that DWI volume at diagnosis is negatively associated with uric acid levels at day 3 and 4. There were no significant associations between total antioxidant capacity of plasma and DWI at diagnosis, or FLAIR at any point.

**Discussion:**

Patients with larger infarcts exhibited a significant decrease in serum uric acid levels, accounting for a more prominent reactive oxygen species scavenging activity with subsequent consumption and decay of this antioxidant. The different kinetics of total antioxidant capacity of plasma and serum uric acid levels suggests a specific role of uric acid in the antioxidant response in ischemic stroke.

## Introduction

Uric acid is an endogenous extracellular antioxidant derived from the purine metabolism, that accounts for about 70% of total antioxidant capacity of plasma [1]. In the last decade it has gained considerable attention in stroke as a potential neuroprotective agent, however, depending on the chemical microenvironment it can also behave as a pro-oxidant [2]. Therefore, the definitive role of uric acid in the pathophysiology of ischemic stroke remains poorly understood.

Comprehensive epidemiological studies have demonstrated the association of high levels of serum uric acid with an increased risk of ischemic stroke [3,4], as well as other cardiovascular conditions [5]. In addition, uric acid has also been reported to be lower in patients with acute ischemic stroke [6], and to exert neuroprotective effects, both in animal models of ischemic stroke [7,8], and in stroke patients [9,10]. Recently, Liu and colleagues reported that patients with a poor outcome had significantly lower uric acid levels than those with a better outcome [11].

It is of interest to note that most studies have been focused on single uric acid measurements [9,10,12]. In a seminal paper of antioxidants in acute ischemic stroke, Cherubini and colleagues reported reduced levels of uric acid in stroke patients, with subsequent normalization after 1 week [6]. On the other hand, in two longitudinal studies, uric acid declined after stroke, reaching the nadir at 6 h [13] and at 48 hours after symptoms onset [14]. Moreover, in stroke patients treated with thrombolytic therapy, Amaro and colleagues described a negative correlation between uric acid during day 1 and the volume of infarction on computed tomography (CT) scan 24 hours after thrombolytic therapy [10]. However, the relationship bewteen a serial evaluation of uric acid levels during the acute phase of stroke and the final infarct volume has not yet been determined.

The current study was aimed to characterize the time course of changes in acid uric levels during acute phase of ischemic stroke and associate them with the final infarct size.

## Methods

### Patients

We performed a cohort study of patients with confirmed diagnosis of ischemic stroke, admitted to the Clínica Santa María Hospital (Santiago, Chile) between May 2014 and July 2016. Written informed consent was obtained from each patient. The study was approved by the ethics committee of the Clínica Santa María Hospital in 2014. A total of 32 patients over 18 years-old, with ischemic stroke confirmed by Magnetic Resonance Imaging (MRI) within 24 hours from onset of symptoms, presenting a baseline National Institute of Health Stroke Scale (NIHSS) <23, which were not candidates for thrombolysis according to current criteria at the time (intravenous, mixed, intra-arterial or mechanical) were included. We excluded patients with lacunar infarcts. We also excluded patients with history of chronic kidney disease (creatinine >2.0 mg/dL), chronic liver disease, uncontrolled diabetes (ketoacidosis or hyperosmolar coma), sepsis or evidence of intracranial haemorrhage on the CT or MRI. A neurologist conducted neurological examination of patients in the emergency room, and subsequently every day during the first week. Daily blood samples were drawn during the first week after symptoms onset or until discharge, and an additional 30-day sample was obtained.

### Neuroimaging

Imaging assessment of vessel occlusion by CT or MR angiography was performed by an experienced stroke neuroradiologist blind to pre-existing clinical and imaging information, allowing to classify patients in two groups: (i) with an occluded vessel (OV) and (ii) with non-occluded vessel (NOV) at the diagnosis. We obtained a diffusion weighted image (DWI) and fluid attenuation inversion recovery (FLAIR) sequences at diagnosis and a 30-day FLAIR MRI sequence. Volumetric analysis of infarcts was quantitatively performed using the Osirix open source DICOM viewer. We used Osirix closed polygon tool to manually outline the infarct areas, thereby creating a region of interest for each sequence.

### Biochemical measurements

Uric acid was determined using standard laboratory techniques, with urate oxidase reagent on an automatic analyzer. Total antioxidant capacity of plasma was evaluated through the ferric reducing ability of plasma (FRAP), and it was measured by spectrophotometry at 593 nm with a sensitivity of 10 μM, as previously described [15].

### Statistical analyses

The continuous quantitative variables are expressed as mean and standard deviation (SD) and discrete quantitative variables are expressed as median and interquartile range. To evaluate the evolution of plasmatic antioxidant levels we performed a mixed model analysis of uric acid and FRAP values during follow-up, adjusted by sex, age and DWI volume at diagnosis. The days were treated as dummy variables, and day 1 was used as the reference. We used multivariable linear regressions of uric acid values during follow up on DWI volumes, and of FLAIR at 30 days on uric acid values during follow up. Confidence intervals of 95% were used and p-values of 0.05 were considered significant, nonetheless, p-values between 0.05 and 0.1 are highlighted and discussed. Data were analyzed using STATA version 15.0

## Results

### Demographics and baseline characteristics

Thirty-two patients were enrolled in the study. One patient was excluded from the analysis during follow up because a diagnosis of sepsis. Demographic characteristics of the sample are presented in **Table 1**. Patients were predominantly males (58.1%), with a mean age of 60.6 years old. The mean time to consultation was 386.7 min. Most of the infarcts were due to occlusion in the middle cerebral artery (MCA) territory, and 18 patients (58.1%) presented with an OV at the diagnosis. Median NIHSS score at diagnosis was 3 points (IQR 2–6). The involved territories and the location of occluded vessels at diagnosis are presented in **Table 1.**

**Table 1:** Clinical characteristics of stroke patients. MCA M1 = M1 Segment of the middle cerebral artery; PCA = Posterior cerebral artery; PICA = Postero-inferior cerebellar artery.

Variable		n = 31
Sex female, n (%)		13 (41.2)
Age—years, mean (SD)		60.6 (17.6)
Comorbidites, n (%)		
	High Blood Pressure	19 (61.3)
	Diabetes	7 (22.6)
	Dislipidemy	10 (32.3)
	Smokers	10 (32.3)
	Atrial Fibrillation	3 (9.7)
	Structural cardiopathy	20 (64.5)
Time to consultation, mean (SD)		386.7 (262.9)
Infarct localization, n (%)		
	Anterior circulation	21 (67.7)
	Posterior circulation	10 (32.3)
	DWI volume at diagnosis (ml), median (IQR)	12.17 (5.105–40.39)
	FLAIR volume at 30 days (ml), median (IQR)	8.1 (2.4–23.2)
Occluded vessel, n (%)		18 (58.1)
MCA M1, n		4
MCA M2-M3, n		9
PCA, n		4
PICA, n		1
NIHSS day 1, median (IQR)		3 (2–6)
Modified Rankin scale ≤2 at day 30, (%)		92.6

### Oxidative stress biomarkers profile during the first week of stroke

**Fig 1:**
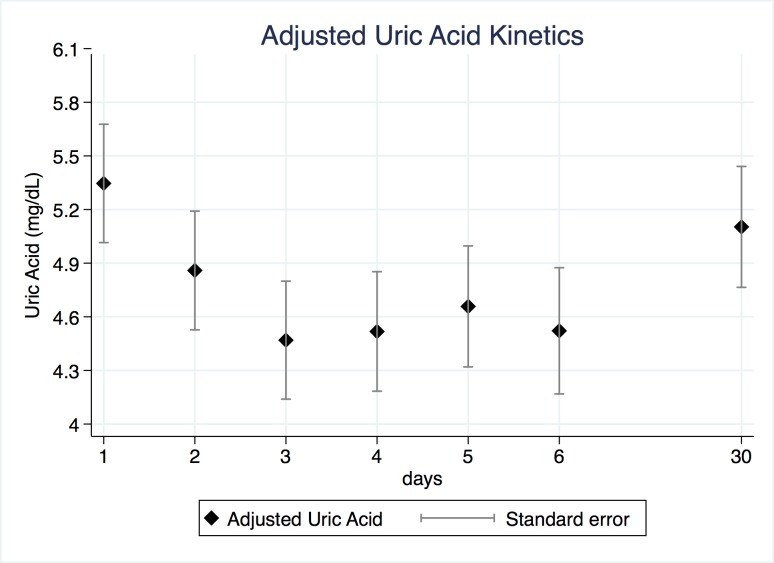
Adjusted uric acid kinetics. Adjusted values of uric acid during the first week after symptoms onset and 30 days later. Diamonds represent uric acid adjusted by sex, age and DWI volume at diagnosis, and data dispersion is presented as standard error.

**Fig 2:**
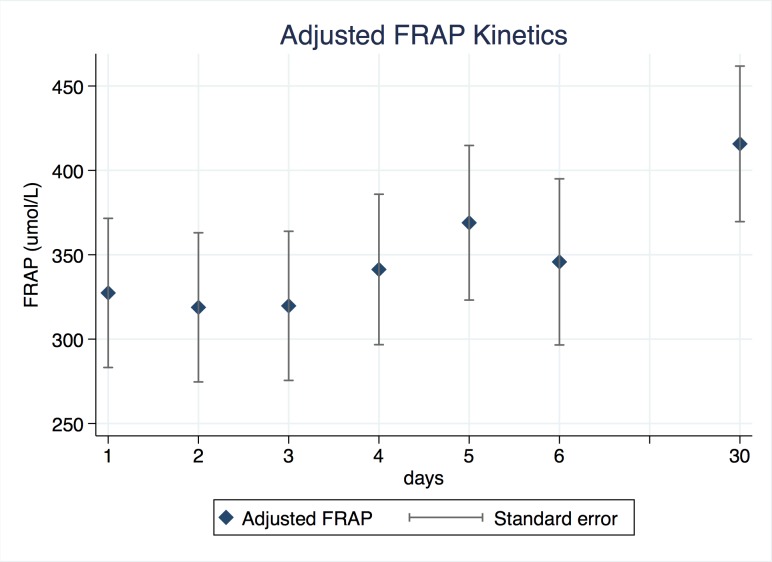
Adjusted FRAP kinetics. Adjusted values of FRAP during the first week after symptoms onset and 30 days later. Diamonds represent uric acid adjusted by sex, age and DWI volume at diagnosis, and data dispersion is presented as standard error.

Uric acid significantly decreased between days 2 to 6 compared to day 1, after adjustment by sex, age and DWI at diagnosis (**Table 2**). The 30-days level of uric acid was not different to day 1. DWI volume is negatively associated with uric acid (p = 0.041). From this model, we estimated the adjusted mean values of uric acid and standard error for each day, as shown in **Fig 1.** Conversely, FRAP levels remained steady between day 1 to day 6, with a delayed increase at day 30 (**Table 3**). We estimated the adjusted mean values of FRAP and standard errors, as shown in **Fig 2**.

**Table 2:** Mixed model analysis for repeated measures of uric acid during follow up. * = significant.

	Coefficient	Std. Error	P value	[95% Conf. Interval]
Day 2 (mg/dL)	-0.487	0.177	*0.006	-0.833 to -0.141
Day 3 (mg/dL)	-0.877	0.173	*0.000	-1.216 to -0.538
Day 4 (mg/dL)	-0.828	0.181	*0.000	-1.183 to -0.473
Day 5 (mg/dL)	-0.688	0.188	*0.000	-1.056 to -0.319
Day 6 (mg/dL)	-0.825	0.213	*0.000	-1.241 to -0.408
Day 30 (mg/dL)	-0.243	0.187	0.195	-0.61 to 0.124
Age (mg/dL per year)	-0.009	0.009	0.313	-0.028 to 0.009
Male Sex (mg/dL)	1.877	0.343	0.000	1.204 to 2.55
DWI (mg/dL per ml of infarct)	-0.01	0.005	0.041	-0.020 to -0.001
Constant (mg/dL)	5.067	0.674	0.000	3.746 to 6.389

**Table 3:** Mixed model analysis for repeated measures of FRAP during follow up. * = significant.

	Coefficient	Std. Error	P value	[95% Conf. Interval]
Day 2 (mg/dL)	-8.546	27.886	0.759	-63.203 to 46.11
Day 3 (mg/dL)	-7.656	27.886	0.784	-62.313 to 47
Day 4 (mg/dL)	13.909	28.504	0.626	-41.958 to 69.776
Day 5 (mg/dL)	41.583	30.319	0.170	-17.841 to 101.007
Day 6 (mg/dL)	18.392	35.073	0.600	-50.35 to 87.135
Day 30 (mg/dL)	88.326	30.741	*0.004	28.075 to 148.577
Age (mg/dL per year)	-0.56	1.205	0.642	-2.922 to 1.801
Male Sex (mg/dL)	38.748	44.536	0.384	-48.542 to 126.038
DWI (mg/dL per ml of infarct)	-0.681	0.649	0.295	-1.953 to 0.592
Constant (mg/dL)	356.545	87.964	0.000	184.14 to 528.951

### Association between serum oxidative stress biomarkers and infarct volumes

**Fig 3:**
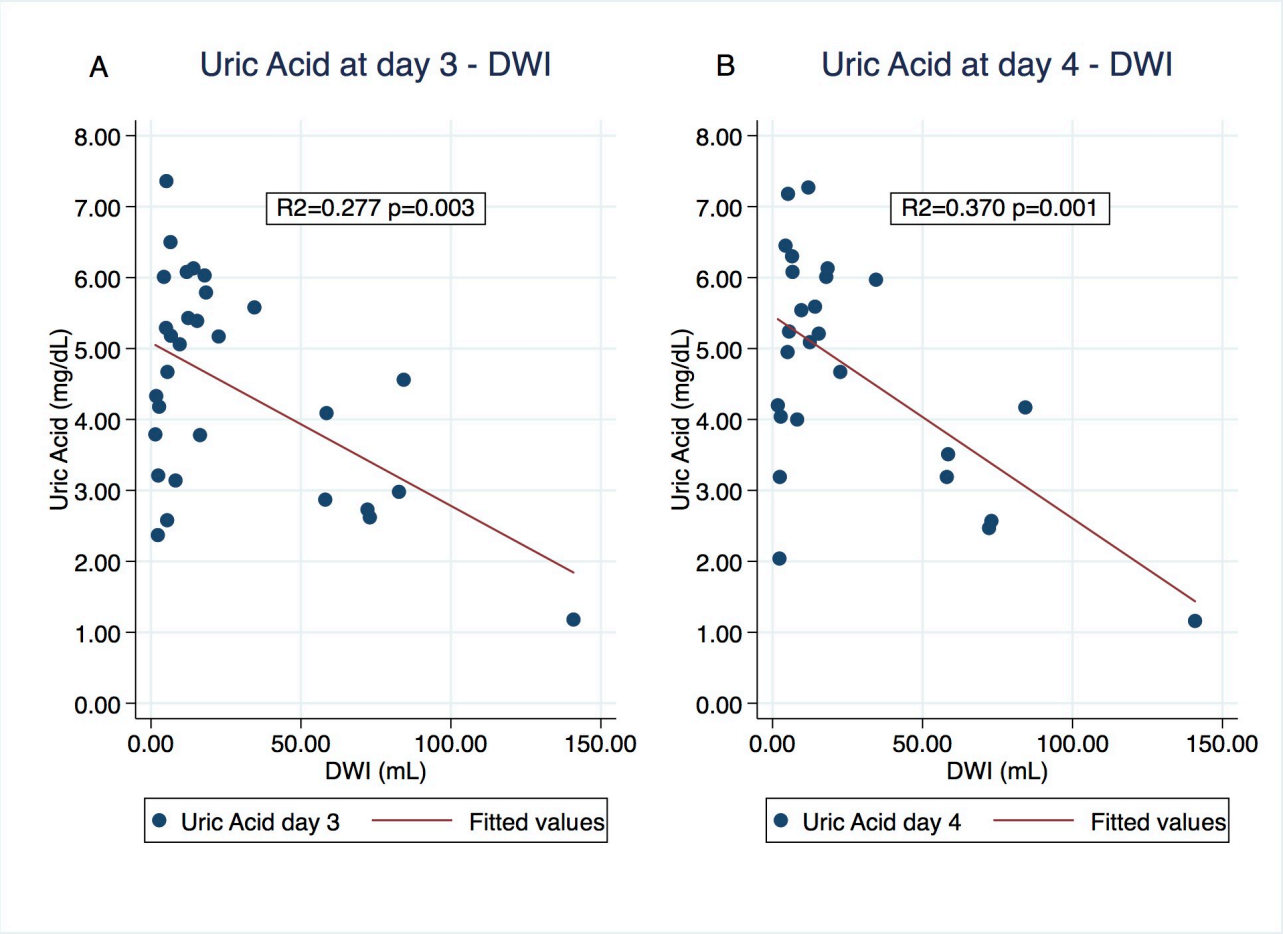
Linear regression of crude values of uric acid at day 3 (A) and day 4 (B) on DWI at diagnosis.

We performed a multivariable linear regression of uric acid values during follow up on DWI volumes, adjusted by sex and age. DWI volume at diagnosis is negatively associated with uric acid levels at day 3 and 4. We present the estimated coefficient, standard error, R-squared and the p value for each case in **Table 4**. Additionally, we graphed the linear regression of crude values for uric acid at day 3 and day 4 in **Fig 3A and 3B** (coefficient 0.023, R-squared 0.277, p value 0.003 and coefficient 0.029 R-squared 0.370, p value 0.001, respectively).

**Table 4:** Multivariable linear regression of uric acid values during follow up on DWI volumes, adjusted by sex and age. UA = Uric acid, * = significant.

	Coefficient	Std. Error	P value	[95% Conf. Interval]	R-squared
UA1-DWI	0.003	0.01	0.763	-0.017 to 0.023	0.245
UA2-DWI	-0.008	0.006	0.183	-0.02 to 0.004	0.467
UA3-DWI	-0.014	0.005	*0.008	-0.025 to -0.004	0.687
UA4-DWI	-0.02	0.006	*0.003	-0.032 to -0.008	0.693
UA5-DWI	-0.012	0.008	0.14	-0.028 to 0.004	0.572
UA6-DWI	-0.007	0.008	0.426	-0.027 to 0.012	0.585
UA30-DWI	-0.003	0.011	0.789	-0.026 to 0.02	0.36

We also performed a multivariable linear regression of FLAIR at day 30 on uric acid values during follow up, adjusted by sex, age and NIHSS score. Here, we found a borderline significant association between FLAIR volume at day 30 and uric acid at days 3 and 4. We present the estimated coefficient, standard error, R-squared and the p value for each case (**Table 5)**.

**Table 5:** Multivariable linear regression of FLAIR at day 30 on uric acid values during follow up, adjusted by sex, age and NIHSS score. UA = Uric acid.

	Coefficient	Std. Error	P value	[95% Conf. Interval]	R-squared
FLAIR30-UA1	-0.052	3.855	0.989	-8.094 to 7.99	0.299
FLAIR30-UA2	1.672	6.003	0.784	-10.941 to 14.285	0.294
FLAIR30-UA3	-10.286	5.469	0.075	-21.694 to 1.122	0.404
FLAIR30-UA4	-9.055	4.881	0.079	-19.271 to 1.162	0.214
FLAIR30-UA5	-6.717	5.119	0.212	-17.776 to 4.342	0.244
FLAIR30-UA6	1.747	11.629	0.885	-25.751 to 29.244	0.214
FLAIR30-UA30	1.38	4.936	0.783	-8.991 to 11.75	0.087

Subgroup analysis did not evidence any significant differences according to vessel occlusion status at diagnosis. There were no significant associations between FRAP levels and DWI at diagnosis, or FLAIR at any point.

## Discussion

Different circumstances determine that the central nervous system is a highly vulnerable organ to oxidative damage, including high concentrations of peroxidisable lipids, low levels of protective antioxidants, low activity of antioxidant enzymes, high oxygen consumption, and high levels of iron acting as pro-oxidants under pathological conditions [16,17]. During an oxidative challenge, such as that occurring during the acute phase of stroke, cellular endogenous antioxidant mechanisms remove reactive oxygen species (ROS) or even prevent their generation. Enzymatic and non-enzymatic antioxidants such as vitamin C, vitamin E and uric acid, contribute to the antioxidant capacity of the central nervous system, mainly by acting as ROS scavengers [18].

The present study demonstrates for the first time that higher volumes of DWI on MRI at diagnosis are associated to lower serum uric acid levels in stroke patients during the first week after symptoms onset (**Table 2** and **Fig 1**). Moreover, multivariable linear regressions of uric acid at third and fourth days on DWI volumes at diagnosis demonstrated a significant negative association, suggesting that initial infarct size is a predictor of uric acid levels at day 3 and 4 (**Table 4** and **Fig 3**). This finding was significant after the adjustment by sex and age.

Our results are in line with those of the URICO-Ictus trial as we found a significant reduction of uric acid levels in the acute phase of ischemic stroke, in this case after adjustment by sex, age, and DWI volume at diagnosis [14,19]. The kinetics of uric acid shows that nadir level occurs at 72h, suggesting that uric acid consumption impacts serum levels in a delayed fashion. This may be due to the effects of several pathological processes occurring in the damaged brain tissue such as ROS production, the rate of uric acid consumption and tissue perfusion changes, among others. Therefore, it should be expected that a larger volume of damaged brain tissue associates with a greater uric acid diminution after 72h, a view consistent with the present data.

Our results show a delayed nadir of uric acid compared to previous studies (6h and 48h) [13,14], which is likely related to the fact that our patients did not receive thrombolytic therapy and 58.1% presented with an occluded vessel at diagnosis, therefore if reperfusion occurred it was spontaneously at a later stage.

Rapid blood flow restoration is one of the main responsible for increased ROS generation after ischemia-reperfusion injury, increasing the levels of peroxynitrite mainly through NADPH oxidase activation, among other mechanisms [20]. Uric acid is a natural peroxynitrite scavenger, and a sustained vessel occlusion with a more pronounced and delayed reperfusion damage are likely to be related with both a more significant decrease in uric acid levels, and larger infarct volumes.

Moreover, the negative association between uric acid at day 3 and day 4 and FLAIR volume at day 30 (p = 0.075 and 0.079, respectively), suggests that a lower availability of uric acid impairs the antioxidant response to the ischemic injury, thus contributing to a larger final infarct size. These findings are in line with the attempts of adding uric acid as part of the acute treatment of ischemic stroke [13,14,19].

Conversely, total antioxidant capacity of plasma was not associated with DWI, or FLAIR volume at diagnosis or 30 days. The different kinetics of FRAP showing steady values during the first week suggests a specific role of uric acid in the antioxidant response in ischemic stroke. The increased levels of FRAP at day 30 might be related to a slow systemic antioxidant response regulated at a genomic level, possibly via the Nrf2-ARE axis [21]. Further studies are needed to evaluate this response.

In the last few years, endovascular therapies in addition to thrombolysis have shown clinical benefits in selected stroke patients with a time window up to 24 hours following symptoms onset [22,23]. In a clinical trial, Chamorro and colleagues [14] recently reported that the addition of intravenous uric acid to thrombolytic therapy did not improve neurological outcome at 90 days, although no additional adverse events were described. Moreover, a tertiary analysis of URICO-ICTUS Trial [19] demonstrated a lower incidence of early ischemic worsening in patients with good collaterals treated with uric acid compared with placebo.

The findings of the present study suggest that interventions aiming at reinforcing antioxidant responses after ischemic stroke may have a more significant effect on patients with large volumes of DWI on diagnostic MRI and those who are not candidates for thrombolytic therapies. Moreover, considering the time course of uric acid decline, an antioxidant therapeutic strategy is thought to be effective over a longer time frame compared to reperfusion strategies, increasing the pool of eligible patients. The impact of recanalization after endovascular treatments on the time course of reperfusion injury and ROS scavenging by uric acid needs to be further evaluated.

## Supporting information

S1 Table Clinical, laboratory and imagenologic data of patients. (XLSX)

## References

[1] Becker BF. Towards the physiological function of uric acid. Free Radic Biol Med. 1993;14: 615–631. 8325534 10.1016/0891-5849(93)90143-i

[2] So A, Thorens B. Uric acid transport and disease. J Clin Invest. 2010;120: 1791–1799. 10.1172/JCI42344 20516647 PMC2877959

[3] Storhaug HM, Norvik JV, Toft I, Eriksen BO, Løchen M-L, Zykova S, et al Uric acid is a risk factor for ischemic stroke and all-cause mortality in the general population: a gender specific analysis from The Tromsø Study. BMC Cardiovasc Disord. 2013;13: 115 10.1186/1471-2261-13-115 24330812 PMC4029378

[4] Li M, Hou W, Zhang X, Hu L, Tang Z. Hyperuricemia and risk of stroke: a systematic review and meta-analysis of prospective studies. Atherosclerosis. 2014;232: 265–270. 10.1016/j.atherosclerosis.2013.11.051 24468137

[5] Fang J, Alderman MH. Serum uric acid and cardiovascular mortality the NHANES I epidemiologic follow-up study, 1971–1992. National Health and Nutrition Examination Survey. JAMA. 2000;283: 2404–2410. 10.1001/jama.283.18.2404 10815083

[6] Cherubini A, Polidori MC, Bregnocchi M, Pezzuto S, Cecchetti R, Ingegni T, et al Antioxidant profile and early outcome in stroke patients. Stroke. 2000;31: 2295–2300. 11022053 10.1161/01.str.31.10.2295

[7] Yu ZF, Bruce-Keller AJ, Goodman Y, Mattson MP. Uric acid protects neurons against excitotoxic and metabolic insults in cell culture, and against focal ischemic brain injury in vivo. J Neurosci Res. 1998;53: 613–625. 10.1002/(SICI)1097-4547(19980901)53:5<613::AID-JNR11>3.0.CO;2-1 9726432

[8] Romanos E, Planas AM, Amaro S, Chamorro A. Uric acid reduces brain damage and improves the benefits of rt-PA in a rat model of thromboembolic stroke. J Cereb Blood Flow Metab Off J Int Soc Cereb Blood Flow Metab. 2007;27: 14–20. 10.1038/sj.jcbfm.9600312 16596120

[9] Chamorro A, Obach V, Cervera A, Revilla M, Deulofeu R, Aponte JH. Prognostic significance of uric acid serum concentration in patients with acute ischemic stroke. Stroke. 2002;33: 1048–1052. 11935059 10.1161/hs0402.105927

[10] Amaro S, Urra X, Gómez-Choco M, Obach V, Cervera A, Vargas M, et al Uric acid levels are relevant in patients with stroke treated with thrombolysis. Stroke. 2011;42: S28–32. 10.1161/STROKEAHA.110.596528 21164140

[11] Liu H, Reynolds GP, Wang W, Wei X. Lower uric acid is associated with poor short-term outcome and a higher frequency of posterior arterial involvement in ischemic stroke. Neurol Sci Off J Ital Neurol Soc Ital Soc Clin Neurophysiol. 2018;39: 1117–1119. 10.1007/s10072-018-3307-4 29511962

[12] Mapoure YN, Ayeah CM, Doualla MS, Ba H, Ngahane HBM, Mbahe S, et al Serum Uric Acid Is Associated with Poor Outcome in Black Africans in the Acute Phase of Stroke. Stroke Res Treat. 2017;2017: 1935136 10.1155/2017/1935136 29082062 PMC5610810

[13] Amaro S, Soy D, Obach V, Cervera A, Planas AM, Chamorro A. A pilot study of dual treatment with recombinant tissue plasminogen activator and uric acid in acute ischemic stroke. Stroke. 2007;38: 2173–2175. 10.1161/STROKEAHA.106.480699 17525395

[14] Chamorro A, Amaro S, Castellanos M, Segura T, Arenillas J, Martí-Fábregas J, et al Safety and efficacy of uric acid in patients with acute stroke (URICO-ICTUS): a randomised, double-blind phase 2b/3 trial. Lancet Neurol. 2014;13: 453–460. 10.1016/S1474-4422(14)70054-7 24703208

[15] Benzie IF, Strain JJ. The ferric reducing ability of plasma (FRAP) as a measure of “antioxidant power”: the FRAP assay. Anal Biochem. 1996;239: 70–76. 10.1006/abio.1996.0292 8660627

[16] Saeed SA, Shad KF, Saleem T, Javed F, Khan MU. Some new prospects in the understanding of the molecular basis of the pathogenesis of stroke. Exp Brain Res Exp Hirnforsch Expérimentation Cérébrale. 2007;182: 1–10. 10.1007/s00221-007-1050-9 17665180

[17] Rodrigo R, Fernández-Gajardo R, Gutiérrez R, Matamala JM, Carrasco R, Miranda-Merchak A, et al Oxidative stress and pathophysiology of ischemic stroke: novel therapeutic opportunities. CNS Neurol Disord Drug Targets. 2013;12: 698–714. 23469845 10.2174/1871527311312050015

[18] Patel M. Targeting oxidative stress in central nervous system disorders. Trends Pharmacol Sci. 2016;37: 768–778. 10.1016/j.tips.2016.06.007 27491897 PMC5333771

[19] Amaro S, Laredo C, Renú A, Llull L, Rudilosso S, Obach V, et al Uric Acid Therapy Prevents Early Ischemic Stroke Progression: A Tertiary Analysis of the URICO-ICTUS Trial (Efficacy Study of Combined Treatment With Uric Acid and r-tPA in Acute Ischemic Stroke). Stroke. 2016;47: 2874–2876. 10.1161/STROKEAHA.116.014672 27758945

[20] Abramov AY, Scorziello A, Duchen MR. Three distinct mechanisms generate oxygen free radicals in neurons and contribute to cell death during anoxia and reoxygenation. J Neurosci Off J Soc Neurosci. 2007;27: 1129–1138. 10.1523/JNEUROSCI.4468-06.2007 17267568 PMC6673180

[21] Kobayashi M, Yamamoto M. Molecular mechanisms activating the Nrf2-Keap1 pathway of antioxidant gene regulation. Antioxid Redox Signal. 2005;7: 385–94. 10.1089/ars.2005.7.385 15706085

[22] Touma L, Filion KB, Sterling LH, Atallah R, Windle SB, Eisenberg MJ. Stent Retrievers for the Treatment of Acute Ischemic Stroke: A Systematic Review and Meta-analysis of Randomized Clinical Trials. JAMA Neurol. 2016;73: 275–281. 10.1001/jamaneurol.2015.4441 26810499

[23] Vidale S, Longoni M, Valvassori L, Agostoni E. Mechanical Thrombectomy in Strokes with Large-Vessel Occlusion Beyond 6 Hours: A Pooled Analysis of Randomized Trials. J Clin Neurol Seoul Korea. 2018;14: 407–412. 10.3988/jcn.2018.14.3.407 29971982 PMC6032006

